# A novel framework for enhancing transparency in credit scoring: Leveraging Shapley values for interpretable credit scorecards

**DOI:** 10.1371/journal.pone.0308718

**Published:** 2024-08-12

**Authors:** Rivalani Hlongwane, Kutlwano Ramabao, Wilson Mongwe

**Affiliations:** 1 Graduate School of Business, University of Cape, Cape Town, South Africa; 2 Electrical and Electronic Engineering, University of Johannesburg, Johannesburg, South Africa; MIT World Peace University Faculty for Engineering and Technology, INDIA

## Abstract

Credit scorecards are essential tools for banks to assess the creditworthiness of loan applicants. While advanced machine learning models like XGBoost and random forest often outperform traditional logistic regression in predictive accuracy, their lack of interpretability hinders their adoption in practice. This study bridges the gap between research and practice by developing a novel framework for constructing interpretable credit scorecards using Shapley values. We apply this framework to two credit datasets, discretizing numerical variables and utilizing one-hot encoding to facilitate model development. Shapley values are then employed to derive credit scores for each predictor variable group in XGBoost, random forest, LightGBM, and CatBoost models. Our results demonstrate that this approach yields credit scorecards with interpretability comparable to logistic regression while maintaining superior predictive accuracy. This framework offers a practical and effective solution for credit practitioners seeking to leverage the power of advanced models without sacrificing transparency and regulatory compliance.

## Introduction

Banks play a crucial role in the economy, influencing the financial landscape while making critical lending decisions that balance risk and profitability for both individuals and businesses [1–3]. To mitigate losses and identify low-risk applicants, banks rely on credit scoring models, or credit scorecards, that use predictor variables to generate credit scores [4]. Accurate identification of high-risk applicants is essential for effective lending, and regulatory frameworks often mandate that credit decisions, especially loan rejections, be transparent and explainable [5–7]. Traditional credit scorecards achieve this transparency through interpretable models like logistic regression [8].

Despite the dominance of logistic regression in credit scoring due to its simplicity and interpretability, recent research has highlighted the superior accuracy of tree-based models such as eXtreme gradient boosting (XGBoost) and random forest [5, 9, 10]. However, their limited interpretability poses significant challenges in practical application, particularly in the banking sector [11]. The "black box" nature of these models makes it difficult for practitioners to understand the underlying reasons behind credit decisions, hindering regulatory compliance, model validation, and effective communication with customers [6, 7, 11].

Specific challenges with tree-based models include:

- Regulatory Compliance: Banks are required to provide clear reasons for loan rejections. The opaque nature of tree-based models complicates this requirement [6, 7].
- Model Validation: The lack of transparency makes it difficult for banks to validate and trust the models, which is crucial for deployment in a highly regulated industry [11].
- Customer Communication: Banks need to explain credit decisions to customers in an understandable manner. The complexity of tree-based models hampers this communication [11].

While the SHapley Additive exPlanations (SHAP) framework, leveraging Shapley values, has been proposed to enhance the interpretability of these models, initially for XGBoost [12], the focus has primarily been on probabilities rather than the credit scores used by credit practitioners [4]. This discrepancy between research advancements and practical needs underscores the importance of developing methods that can harness the predictive power of advanced models while ensuring the transparency and interpretability required in the banking sector.

This research aims to bridge this gap by demonstrating how Shapley values derived from tree-based models like XGBoost and random forest can be used to generate credit scores that are comparable to those from logistic regression-based credit scorecards, using two credit datasets. Additionally, we explore how these Shapley value-derived scores align with current practices for explaining credit decisions. By combining accuracy with interpretability, this study aims to promote the adoption of transparent and high-performing models in practical credit scoring, empowering banks to make informed lending decisions.

The paper is organized as follows: We begin with an overview of key themes in credit risk modelling, examining logistic regression, advanced scoring models, and the imperative aspect of interpretability. Following this, we describe the methodological approach used in this research, present our findings and results, and conclude with insights into future research directions.

## Literature review

This section offers a comprehensive overview of key themes in credit risk modelling. It examines logistic regression, advanced scoring models, and the imperative aspect of interpretability. This review not only traces the historical significance of logistic regression and advanced models but also underscores the evolving challenges and solutions tied to model interpretability. Through this exploration, the section lays the groundwork for a deeper understanding of credit risk assessment methodologies.

### Credit scoring models

Logistic regression, a technique with roots in the 19th century [13], is the most common credit risk model in practice due to its simplicity and ability to produce interpretable predictions [4, 5, 8, 14]. Its prominence and scope of application expanded following research by [15, 16], with early examples of its use in credit risk seen in the work of [17]. A study by [18] comparing five base learners on a credit loan dataset found that logistic regression outperformed decision trees, naïve bayes, and AdaBoost in terms of AUC and accuracy metrics, but was surpassed by random forest and XGBoost. This highlights the trade-offs between interpretability and performance in credit risk modelling.

The logistic regression model’s structure facilitates this interpretability by relating predictor variables to the probability of an event (such as default) through a logit transformation. The model consists of an additive component, the sum of the intercept and a product of model parameters and their respective predictor variables [19]. The intercept represents the average value of the natural log of the odds when the predictor variables equal zero [19]. The logistic regression model is expressed as follows [19]:

$$\ln\left(odds\right)=ln\left(\frac{p}{1-p}\right)=\beta_{0}+\sum_{i=1}^{m}\beta_{i}x_{i}$$ (1)

where *β*_0_ is the intercept, *β_i_*, *i* = 1,2,…*m* are parameters of the predictor variables *x_i_*, *i* = 1,2,…*m*.

Research in credit risk modelling remains active, with a strong focus on improving the accuracy of models, particularly through tree-based methods [5]. Studies have shown that tree-based models, such as XGBoost, random forest, LightGBM, and CatBoost, often outperform traditional models like logistic regression in terms of accuracy [5]. These models construct numerous non-linear decision trees by iteratively selecting subsets of data, with XGBoost further employing a boosting technique to combine multiple weak learners and enhance predictive accuracy [20].

Tree-based models make predictions through majority voting, where the final prediction is based on the most frequent outcome among the individual trees [20, 21]. This approach, as demonstrated in various studies [14, 20, 21], often leads to superior predictive performance. Notably, [14] indicated that XGBoost handles imbalanced datasets—a common characteristic of credit data due to the rarity of defaults compared to non-defaults [22]—better than other advanced scoring methods.

Despite their popularity in research [4, 5] and superior prediction accuracy compared to logistic regression [5], tree-based models remain less common in practical credit scoring [11]. This is largely due to their inherent complexity, which makes it difficult to interpret their predictions and explain the reasons behind credit decisions, a crucial requirement in many regulatory contexts.

A 2015 survey of machine learning models used in data science competitions found that XGBoost was the most popular choice, offering higher prediction accuracy in various domains, including credit risk [9]. A benchmarking study on credit data further demonstrated XGBoost’s superior accuracy compared to logistic regression, neural networks, support vector machines, and random forest, even outperforming FICO scores [10].

Similar to XGBoost, LightGBM is a gradient boosting model, but it differs in its depth-first tree growth strategy, often leading to faster performance [23]. Studies [24, 25] have shown LightGBM’s superior predictive performance on credit data compared to XGBoost and CatBoost.

CatBoost, another member of the gradient boosting family, stands out for its handling of categorical variables, making it valuable for datasets where categorical data plays a crucial role in predictive modelling [26]. Research has shown that CatBoost can outperform both XGBoost and LightGBM models in terms of predictive performance on credit data [23].

In random forest models, multiple decision trees are built, and the final prediction is determined through majority voting, where the most common prediction among the trees is selected [27]. Research has shown that tuning hyperparameters, such as the number of trees and predictor variables, is crucial for optimizing random forest performance in credit scoring [28, 29].

Studies comparing the performance of different models in credit scoring have reported varying AUC values. For instance, [30] investigated logistic regression and a neural network, achieving AUC values of 0.711 and 0.731, respectively. The study in [31] obtained an AUC of 0.680 from a random forest model before implementing a data sampling methodology for balancing and achieved higher AUC after applying their proposed technique.

While tree-based models have showcased high accuracy in their predictions, their limited ability to offer human-understandable explanations have constrained their adoption. This challenge has been acknowledged and addressed by [32], yet it continues to hinder the widespread use of these advanced models in real-world credit scoring applications.

### Interpretability

The lack of human-understandable explanations for predictions made by advanced machine learning models is the primary obstacle to their wider adoption in practice [14, 32]. This concern is echoed by credit regulators in the USA and RSA, who require models used for credit decisions to provide human-understandable interpretations [6, 7, 33]. In addition to explaining loan rejections, interpretability is also crucial for communicating low and high credit scores to various stakeholders, including credit practitioners, auditors, regulators, senior management, and model validators [4].

To address this challenge, the SHAP framework, rooted in game theory, was introduced by [12] to enhance the interpretability of machine learning models. Originally developed by [34] to determine the fair distribution of payouts in cooperative games, the SHAP framework calculates Shapley values for each predictor variable in a model [35]. These values represent the marginal contribution of each variable to a prediction and can be used to provide human-understandable explanations for credit decisions, aligning with the requirements outlined by [4].

Researchers have adopted the SHAP framework to provide detailed explanations of complex machine models [36–38], with the motivation of increasing understanding and trust in these models [38]. In the context of credit risk scoring, the SHAP framework has been used to explain predictions made by tree-based gradient boosting models [37, 39, 40].

Studies such as [37, 40] utilized SHAP to compute and compare marginal probabilities of predictor variables in tree-based models, finding significant differences in predictions and highlighting the higher default risk predicted by tree-based models. Similarly, [39] used SHAP with counterfactuals to provide explanations for predictions made by a tree-based gradient boosting model, ultimately concluding that the methodology helps in understanding the model’s behaviour.

While previous studies have explored the use of SHAP for explaining credit scores, they have not addressed how these explanations align with the credit scores used by practitioners, nor how they can be used to identify specific predictor variable categories that lead to lower scores and potential rejections. Our research aims to fill this gap, particularly when using tree-based models, by demonstrating the practical application of Shapley values. Our goal is to empower credit professionals to identify predictor variable categories that substantially impact lower credit scores, potentially resulting in credit application denials. This will ultimately enhance the transparency and effectiveness of credit assessment processes.

### Literature review summary

This section offers a synthesis of the preceding sections, encompassing credit scoring models in practical application and literature. Table 1 provides a condensed overview of how prior research leveraged the SHAP framework to enhance the interpretability of advanced credit scoring models.

**Table 1:** Overview of the literature.

Research Focus	Key Findings
**Credit scoring models**	Logistic regression, acknowledged as the most common credit risk model in practice [4, 5, 8] and with roots tracing back to the 19th century [13], gained prominence through [15, 16]. Its valued attributes encompass simplicity and interpretability, particularly in banking contexts [14]. Notably, it comprises additive components as elucidated in reference [19], thus solidifying its enduring role in credit risk assessment. Advanced Scoring Models, including XGBoost and random forest, exhibit notably higher accuracy compared to logistic regression [5, 20]. Leveraging non-linear trees and boosting [20], they’re prominent in research, yet constrained in practicality due to interpretability [4, 5]. They employ multiple trees with final predictions by majority voting [27], optimized through hyperparameter tuning [28, 29], they’re favoured for precision in credit scoring [28, 29].
**Interpretability using the SHAP framework**	SHAP framework applied to machine models enhances understanding and trust [38]. In emerging credit risk scoring studies, SHAP reveals variable influences [37, 39, 40]. Notably, [37] and [39] extract log-odds and probabilities from SHAP for insights into predictor significance. [40] demonstrates heightened default predictions by gradient boosting models and examines predictor marginal probabilities. SHAP’s efficacy in explaining complex models bolsters predictive superiority [39].

## Methodology

This section outlines the systematic approach employed in this study for credit scoring model development and evaluation. It covers data preprocessing, feature engineering, variable selection, Shapley values integration, credit score computation, encoding methods, data partitioning, hyperparameter tuning, and model performance metrics. This section provides a concise overview of the methodology used to construct and assess the credit scoring models.

### Data

This research employs two datasets: the Taiwan Credit Card data from [41], comprising 30,000 loan accounts (6,636 in default, a 22.12% default rate) from April to September 2005, and the Home Credit data from [42], containing 356,255 customers (24,845 classified as "bad" due to default, a 6.97% default rate), released on Kaggle in June 2018. The Taiwan Credit Card dataset includes 23 predictor variables, encompassing demographics, credit history, payment behaviour, and financial characteristics, while the Home Credit dataset contains 217 variables, including credit bureau, alternative, and demographic data.

To develop the models, both datasets are split into 80% training data and 20% test data using probability-based sampling to ensure consistent results and maintain the independence of the test set [4, 43]. The reported results of the model’s performance are based on the test data. However, this approach has limitations, such as the fixed 80–20 split ratio recommended in [4], which may not be optimal for all datasets and could potentially impact the generalizability of the models.

### Feature engineering

Feature engineering, the process of creating new predictor variables from existing data, can be used to enhance model performance and extract additional insights [44]. This can involve transforming or aggregating variables, as detailed in [44]. In this study, feature engineering was applied to three time-series predictor variables in the Taiwan Credit Card dataset, transforming the original 23 variables into 59. Specifically, we calculated the 3-month rolling average, standard deviation, and the ratio of the current month’s value to the 3-month average for each time-series variable, starting from June and progressing through September.

Data aggregation techniques, including averages, counts, and sums on transactions grouped by client ID, were applied to the Home Credit dataset, expanding the predictor variables from 217 to 767. These aggregations, mirroring the approach in [45], focused on numeric application data, transaction patterns, and timely instalment payment behaviour.

Unlike [30, 31], which used the predictor variables in their raw form, our study leverages these feature-engineered variables, potentially providing a unique perspective on the dataset and its predictive power. This approach may reveal hidden patterns and relationships that could improve the accuracy and interpretability of our credit risk models.

### Variable selection

Permutation importance [46] and the Wald test [47] were employed to reduce the predictor variable set, eliminating variables with minimal contribution to the AUC or lacking statistical significance. This resulted in 7 variables for the Taiwan Credit Card data and 11 variables for the Home Credit data, aligning with recommendations for typical scorecard complexity [48] and mitigating overfitting concerns [49].

Additionally, a correlation analysis following established guidelines [50, 51] assessed multicollinearity. No pairs of predictor variables exceeded the pre-defined 0.8 correlation coefficient threshold [52]. The highest observed correlations were 0.69545 (Home Credit) and 0.75263 (Taiwan Credit Card). These combined steps removed 52 predictor variables from the Taiwan Credit Card data and 756 from the Home Credit data.

Ultimately, for the Home Credit data, this selection process resulted in the predictor variables that are statistically significant, as shown in Table 2.

**Table 2:** Predictor variables for the Home Credit data.

Predictor variable	Description	p-value
AVG_EXT_SOURCE	Average of external scores (1, 2 & 3)	0.04200
EXT_SOURCE_3	Normalized score from external data source	0.00000
EXT_SOURCE_2	Normalized score from external data source	0.00000
EXT_SOURCE_1	Normalized score from external data source	0.00000
BUREAU_DAYS_CREDIT_MAX	Maximum—How many days before current application did client apply for Credit Bureau credit	0.00000
INSTAL_DPD_MEAN	Average—Days past due of Instalments	0.00000
DAYS_EMPLOYED	How many days before the application the person started current employment	0.00000
APPS_ANNUITY_CREDIT_RATIO	Ratio of AMT_ANNUITY / AMT_CREDIT	0.00000
APPROVED_AMT_ANNUITY_MEAN	Average–Approved annuity amount	0.00000
INSTAL_DAYS_ENTRY_PAYMENT_MAX	Maximum number of days (relative to the application date) on which a payment was made for previous instalments	0.00000
APPROVED_AMT_CREDIT_MAX	Maximum–Approved credit amount	0.00000

Similarly, for the Taiwan data, the final list of predictor variables is presented in Table 3.

**Table 3:** Predictor variables for the Taiwan credit data.

Predictor variable	Description	p-value
AVG_PAY__SEP	Average repayment payment status for July, August and September	0.00000
CURRENT_OVER_3MAVG_PAY__SEP	The Ratio of September repayment payment status over Average repayment payment status for July, August and September	0.00000
STD_PAY__SEP	The standard deviation of July, August and September repayment payment status	0.00000
AVG_PAY__JUN	Average repayment payment status for April, May and June	0.00100
AVG_BILL_AMT_SEP	Average bill for July, August and September	0.00000
AVG_PAY_AMT_SEP	Average payment amount for July, August and September	0.00000
CURRENT_OVER_3MAVG_BILL_AMT_SEP	The Ratio of the September bill over Average bill for July, August and September	0.00000

In conclusion, the number of predictor variables in Tables 2 and 3 has been intentionally limited to align with standard credit scorecard development practices, which typically utilize up to 12 variables [48], and to minimize the risk of model overfitting and complexity [49].

### Calculating the credit score in a practice setting

A previous study [4] introduced the concept of a neutral score, the point at which the odds of good and bad outcomes are equal, as a key element in explaining loan application rejections. This score is calculated using parameters such as the intercept of a logistic regression model and the number of predictor variables in the scorecard. The formulas for calculating credit scores, including the neutral score and scores for categorical variables, are well-established and can be found in [4].

Score scaling parameters, offset and factor, are used to adjust the scorecard to achieve desired odds of good to bad outcomes at specific credit score levels. For example, in a logistic regression-based scorecard, a customer’s score falling below the neutral score on a predictor variable is considered a likely reason for credit application decline [4].

While the methodology in [4] provides a foundation for interpretability, our research proposes an alternative approach using Shapley values to further enhance the interpretability of credit scorecards, particularly for tree-based models.

### Shapley values

As indicated earlier, the SHAP framework was proposed by [12] to provide detailed explanations of complex machine learning models through the use of Shapley values. These Shapley values offer three important properties crucial for determining the marginal contribution of each predictor variable in a model [12]:

1. Local Accuracy: Ensures that predictions for a specific instance can be attributed to the input values of each variable for that instance.
2. Missingness: A variable absent from the model does not influence the prediction, similar to how entities that make no contribution in a given context receive no payoffs [53].
3. Consistency: (also known as symmetry) Variables with equal contributions in the model contribute equally to the overall prediction, ensuring fairness and unbiased model performance.

The predictions are given by the following:

$$f(x)=\phi_{0}+\sum_{i=1}^{m}\phi_{i}x_{i}$$ (2)

where *ϕ*_0_ is the naive prediction i.e., prediction without any predictor variables, *ϕ_i_*, *i* = 1,2,…*m* are the parameters of predictor variables *x_i_*, *i* = 1,2,…*m* and *x_i_*, *i* = 1,2,…*m* are the inputs of predictor variables.

### Data processing

Binning, the process of converting continuous variables into categorical ones, is a common practice in credit scoring [4]. It involves grouping values into distinct categories or "bins." This approach simplifies the understanding of relationships between predictor and target variables, streamlines the allocation of credit points, and systematically addresses outliers [4, 54]. It also enhances the ability of banking professionals to derive actionable insights from the data, such as identifying high-risk customer segments or optimal credit score thresholds. In a credit scorecard, each bin is associated with a specific credit score linked to the input values of a predictor variable, allowing for easy comparison with the neutral score and identification of bins where predictor variables fall below the standard [4].

Our binning approach aligns with the standard practice of maximizing the Weight of Evidence (WOE) [4], a measure of the strength of an input value in differentiating between good and bad customers. By discretizing continuous variables into categorical ones, we optimize the WOE metric, ensuring that the resulting bins enhance interpretability and facilitate precise allocation of credit points.

Given that machine learning algorithms like XGBoost require numerical inputs [55], we binned numerical variables and then employed one-hot encoding, a popular and simple method for representing categorical variables [55, 56]. To address missing values in the numerical variables, imputation with the mean of non-missing values was employed for each variable [57]. Additionally, outliers were handled by setting the lower and upper bounds for all observations in each variable to the 2.5th and 97.5th percentiles, respectively [58].

### Hyperparameter tuning

Hyperparameter tuning is essential for optimizing model performance, as it allows for fine-tuning the parameters of ensemble models to achieve superior outcomes [59]. In this study, we employed grid search, a well-established and effective method for finding optimal hyperparameters [60]. Other hyperparameter tuning methods include Bayesian optimization, which uses probabilistic models, random search, which randomly samples hyperparameter combinations, and manual search, guided by human expertise [61, 62]. The choice of method depends on computational resources and problem complexity, as each balances comprehensiveness and efficiency in finding optimal configurations [62].

### Model validation

To validate the models and assess their generalizability, this study employs 5-fold cross-validation, a common technique for estimating machine learning model performance on unseen data [63]. This method involves partitioning the dataset into five subsets (folds), iteratively using each fold as the validation set while the remaining folds are used for training [63]. The process is repeated five times, and the resulting performance metrics are averaged to provide a robust estimate [63]. While effective, k-fold cross-validation can be computationally expensive, particularly for larger values of k [63]. This 5-fold approach aligns with previous studies [23, 25], offering a balance between computational efficiency and model validation rigor.

### Model performance metrics

Most researchers assess the performance of credit scorecards using the AUC [5, 14, 64, 65], due to its ability to indicate a model’s capacity to differentiate between good and bad customers [5]. A higher AUC signifies better discrimination between these two groups [45]. However, AUC has limitations. It can be misleading for poorly fitted models [66] and lacks intuitive interpretation for practitioners [67]. Despite these shortcomings, AUC remains a popular metric in both research and practice [5].

The AUC is calculated as the area under the receiver operating characteristics (ROC) curve, which plots the true positive rate against the false positive rate at various classification thresholds [5]. To assess the statistical significance of differences in AUC between models, we employed the DeLong test [68, 69].

In addition to AUC, misclassification statistics, often presented in a confusion matrix (Table 4), offer a practical way to evaluate credit scorecard performance [4]. This matrix categorizes customers based on their probability of default and compares their actual classification to the scorecard’s prediction, resulting in four cells: true negative, false positive, false negative, and true positive. This comparison helps determine the accuracy of the scorecard’s predictions for good and bad customers.

**Table 4:** Confusion matrix.

		Predicted	
		Good	Bad
**Actual**	Good	True negative	False positive
	Bad	False negative	True positive

To evaluate a credit scorecard’s accuracy, the true negative rate (specificity) measures the model’s ability to predict non-defaulting (good) customers, while the true positive rate (sensitivity) measures its ability to predict defaulting (bad) customers. The aim is to use the scorecard’s probability of default to reduce false positives and false negatives by adjusting the probability cut-off [4].

### Proposed framework for calculating credit scores

This framework outlines a systematic approach for enhancing credit scoring models by integrating Shapley values [12] into the established methodology of [4]. It encompasses the entire process of deriving credit scores, from the initial predictor variable binning to the final credit score calculation. By incorporating Shapley values, this framework provides a comprehensive pathway to derive more transparent and insightful credit scores, ultimately aiding in informed credit decision-making and model refinement.

**Fig 1:**
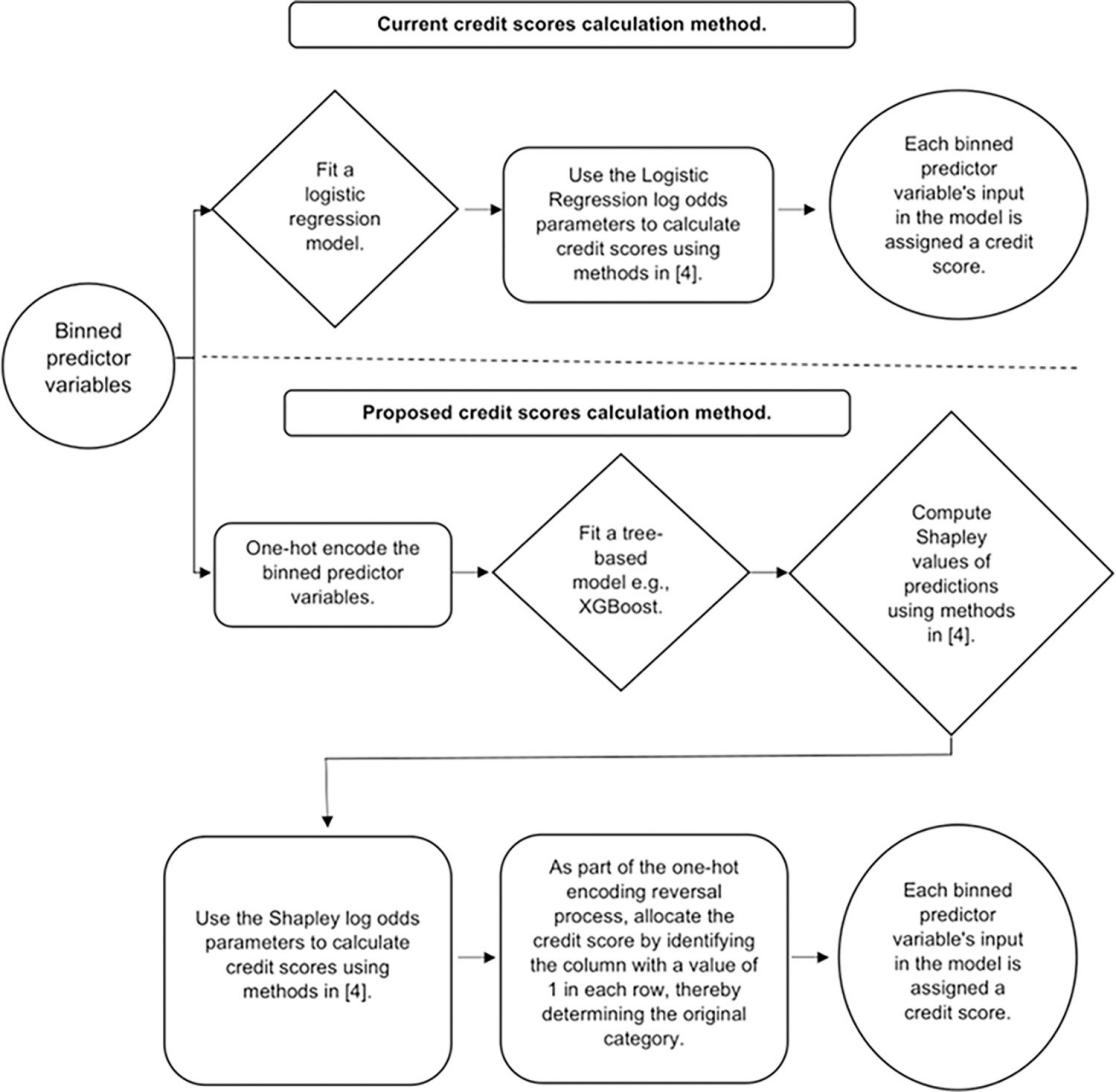
Credit scores calculation process flow—current vs proposed.

Our proposed methodology begins with the binning phase, a crucial step in scorecard development given its significant impact on the final scorecard’s structure [4]. As illustrated in Fig 1, our approach introduces additional stages where one-hot encoding is applied to the binned predictor variables before model fitting, and Shapley values are used in place of logistic regression parameters.

## Results and analysis

This section presents the outcomes of the credit scoring models and delves into their performance. This includes an in-depth examination of credit scorecards associated with each model, illustrating how individual predictor variables are practically represented. Through a detailed exploration of these outcomes, this section offers valuable insights into the effectiveness and real-world applicability of the developed models.

### Performance of the models

Table 5 presents a comparison of the logistic regression, random forest, XGBoost, LightGBM, and CatBoost models in terms of AUC. The random forest model achieved the highest AUC, followed closely by XGBoost and LightGBM. However, the DeLong test [68] indicates that the differences in AUC among these three models are not statistically significant.

**Table 5:** AUC and p-values of the models–Taiwan data.

	p-value				
Model	AUC	XGBoost	LightGBM	Logistic Regression	CatBoost
**Random Forest**	0.75929	0.41580	0.15990	0.00021	0.00143
**XGBoost**	0.75766		0.47520	0.00315	0.00056
**LightGBM**	0.75690			0.00316	0.00310
**Logistic Regression**	0.74891				0.81190
**CatBoost**	0.74793				

Similarly, the AUC values for logistic regression and CatBoost were not significantly different from each other. However, the p-values from the DeLong test show significant differences between the top-performing group (random forest, XGBoost, LightGBM) and the lower-performing group (logistic regression, CatBoost).

Notably, our models outperformed the benchmark AUC of 0.697 reported in previous research [30, 31] that used the same dataset but without applying feature engineering approach. This suggests that feature engineering, which distinguished our study from previous work in terms of predictor variable utilization, contributed to the improved predictive performance.

Table 6 presents the confusion matrices for the Taiwan Credit Card data models, highlighting the superior predictive power of the random forest and XGBoost models. Both achieved the highest overall accuracy (75.717%) and lowest misclassification rate (24.283%), outperforming LightGBM, logistic regression, and CatBoost.

**Table 6:** Confusion matrices of the models–Taiwan data.

Random Forest		Predicted	
		Good	Bad
**Actual**	Good	3,765 (80.363%)	920
	Bad	537	778 (59.163%)
**XGBoost**		**Predicted**	
		Good	Bad
**Actual**	Good	3,767 (80.406%)	918
	Bad	539	776 (59.011%)
**LightGBM**		**Predicted**	
		Good	Bad
**Actual**	Good	3,727 (79.552%)	958
	Bad	522	793 (60.304%)
Logistic Regression		**Predicted**	
		Good	Bad
**Actual**	Good	3,688 (78.719%)	997
	Bad	533	782 (59.468%)
**CatBoost**		**Predicted**	
		Good	Bad
**Actual**	Good	3,657 (78.058%)	1,028
	Bad	515	800 (60.837%)

Table 7 presents the AUC values of the different models on the Home Credit data. The XGBoost model achieved the highest AUC of 0.69766. The DeLong test [68] confirmed that the differences in AUC between XGBoost and all other models, were statistically significant (p-values < 0.05). The only comparison that did not reach statistical significance was between LightGBM and logistic regression, suggesting their AUC values are not significantly different according to the DeLong test [68].

**Table 7:** AUC and p-values of the models–Home Credit data.

	p-value				
Model	AUC	XGBoost	LightGBM	Logistic Regression	CatBoost
**Random Forest**	0.69280	0.00000	0.00044	0.00012	0.00002
**XGBoost**	0.69766		0.02081	0.00466	0.00000
**LightGBM**	0.69654			0.87847	0.00000
**Logistic Regression**	0.69644				0.00000
**CatBoost**	0.68450				

Table 8 presents the confusion matrices of the Home Credit data models. The XGBoost model achieved the highest overall accuracy (70.335%) and the lowest misclassification rate (29.665%) compared to the other models.

**Table 8:** Confusion matrices of the models–Home Credit data.

Random Forest		Predicted	
		Good	Bad
**Actual**	Good	39422 (69.775%)	17077
	Bad	2057	2947 (58.893%)
**XGBoost**		**Predicted**	
		Good	Bad
**Actual**	Good	40299 (71.327%)	16200
	Bad	2045	2959 (59.133%)
**LightGBM**		**Predicted**	
		Good	Bad
**Actual**	Good	40060 (70.904%)	16439
	Bad	2019	2985 (59.652%)
**Logistic Regression**		**Predicted**	
		Good	Bad
**Actual**	Good	39545 (69.992%)	16954
	Bad	2006	2998 (59.912%)
**CatBoost**		**Predicted**	
		Good	Bad
**Actual**	Good	39178 (69.343%)	17321
	Bad	2024	2980 (59.552%)

Overall, these results corroborate previous findings [5, 70] demonstrating the superior performance of tree-based models compared to classic techniques like logistic regression in credit risk assessment.

### Interpretable credit models–Taiwan data

Previous research, such as [37, 39, 40], focused on providing marginal probability or log-odds contributions of each variable in a model, shedding light on their statistical significance.

**Fig 2:**
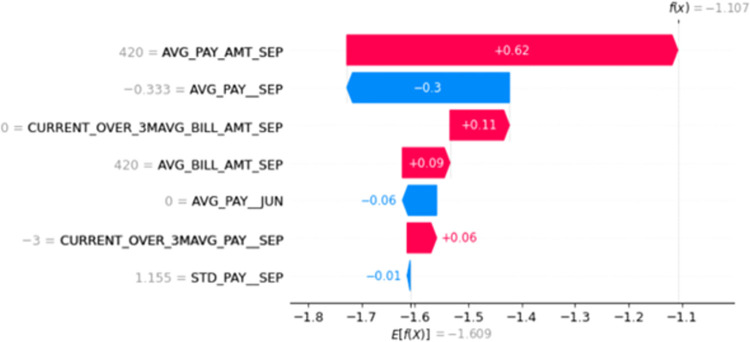
Logodds of the predictor variables.

Fig 2 illustrates the type of interpretability offered by previous studies, showcasing the log-odds contributions of each predictor variable for a specific customer in the dataset. While statistically informative, this type of output, which focuses on log-odds or probabilities, may not be readily interpretable or actionable for credit practitioners who primarily rely on credit scores for decision-making [4]. This section aims to bridge this gap by drawing parallels between the parameters used in logistic regression-based models and those derived from the SHAP framework, proposing to replace logistic regression parameters with Shapley values for identifying top reasons for model predictions. We compare the established method for determining top reasons for credit scorecard predictions [4] with our proposed approach using the SHAP framework [12].

The following representations visually distinguish credit scores below the neutral score by shading them in grey. We provide side-by-side comparisons of credit scores based on both logistic regression parameters and Shapley values. All five models were developed using seven predictor variables with consistent binning.

Tables 9–15 illustrate the credit scores of the predictor variables on the Taiwan data. In most cases, the five models agree regarding the predictor variable bins that lie below the neutral credit score, thereby presenting potential explanations for customers receiving lower credit scores. Except for the predictor variable "Average Bill Amount (July, August, September)" in Table 10, where the random forest model suggests that only the bin (-inf, 13.50) could potentially be cited as a reason for an applicant receiving a lower credit score.

**Table 9:** Average payment indicator—July, August & September.

		Logistic Regression	XGBoost	Random Forest	LightGBM	CatBoost
**Predictor Variable**	Bin	Credit Score				
Average Payment Indicator (July, August, September)	[0.17, inf)	0.0000	77.7914	0.0000	0.0000	0.0000
	(-inf, 0.17)	227.9544	189.8177	203.2322	1145.1521	208.4717
**Neutral Credit Score**		181.5809	167.0278	161.8880	912.1900	166.0616

**Table 10:** Average bill amount—July, August & September.

		Logistic Regression	XGBoost	Random Forest	LightGBM	CatBoost
**Predictor Variable**	Bin	Credit Score				
Average Bill Amount (July, August, September)	(-inf, 13.50)	75.4879	84.5542	69.1873	0.0000	66.7293
	[13.50,49794.83)	119.9583	120.3305	119.6997	106.1236	119.5988
	[49794.83, inf)	154.1544	126.4671	126.5869	129.8170	165.6828
**Neutral Credit Score**		128.18	120.3961	119.2480	107.9997	131.0323

**Table 11:** Average payment indicator–April, May, and June.

		Logistic Regression	XGBoost	Random Forest	LightGBM	CatBoost
**Predictor Variable**	**Bin**	**Credit Score**				
Average Payment Indicator (Apr, May, Jun)	[1.50, inf)	0.0000	33.9752	0.0000	40.5293	0.0000
	[0.50, 1.50)	55.7196	79.5345	0.0000	82.6911	17.5708
	(-inf, 0.50)	172.5623	232.1287	263.2195	1,590.1881	237.1356
**Neutral Credit Score**		151.0169	205.6815	222.4819	1,354.2532	202.0626

**Table 12:** Ratio September payment indicator divided by a 3-months average payment indicator (July, August, and September).

		Logistic Regression	XGBoost	Random Forest	LightGBM	CatBoost
**Predictor Variable**	**Bin**	**Credit Score**				
Ratio September Payment Indicator over a 3 months Average Payment Indicator (July,August, September)	[1.23, inf)	72.9460	18.1803	82.6155	0.0000	0.0000
	[0.20, 1.23)	106.2807	88.8360	109.3608	74.6328	45.9130
	(-inf, 0.20)	171.8272	299.5807	149.1770	264.4279	622.8268
**Neutral Credit Score**		140.2733	201.8103	129.3548	175.2677	370.0489

**Table 13:** Standard deviation of payment indicator—July, August, September.

		Logistic Regression	XGBoost	Random Forest	LightGBM	CatBoost
**Predictor Variable**	**Bin**	**Credit Score**				
Standard Deviation of Payment Indicator (July, August, September)	[0.79, inf)	69.4371	0.0000	93.2310	38.0470	27.2959
	(-inf, 0.79)	155.7639	204.9736	132.7333	219.7735	204.3044
**Neutral Credit Score**		137.4367	161.4577	124.3469	181.1929	166.7255

**Table 14:** Average payment amount—July, August, September.

		Logistic Regression	XGBoost	Random Forest	LightGBM	CatBoost
**Predictor Variable**	**Bin**	**Credit Score**				
Average Payment Amount (July, August, September)	(-inf, 31.17)	62.0784	0.0000	85.1439	0.0000	0.0000
	[31.17,2001.83)	91.5675	7.1893	103.2557	43.4340	0.0000
	[2001.83,4312.17)	131.3691	144.8357	127.4429	141.8376	130.2874
	[4312.17, inf)	181.3795	265.6866	156.7996	246.9170	174.6079
**Neutral Credit Score**		127.3518	121.2622	124.6605	128.1473	86.2906

**Table 15:** Ratio September bill amount over a 3-months average bill amount—July, August, September.

		Logistic Regression	XGBoost	Random Forest	LightGBM	CatBoost
**Predictor Variable**	**Bin**	**Credit Score**				
Ratio September Bill Amount over a 3 months Average Bill Amount (July, August, September)	[0.83, 1.06)	30.9308	91.7615	77.6660	94.3556	0.0000
	(-inf, 0.83)	149.9491	145.6139	127.7371	142.1031	146.3869
	[1.06, inf)	203.8937	191.2323	139.0207	180.9784	193.4898
**Neutral Credit Score**		109.3790	133.7848	107.1574	131.1313	90.5642

The consistency and similarity in predictor variable input values across models have yielded compelling results. The models largely agree on which input values fall below or above the neutral credit score, demonstrating consistency in identifying potential reasons for credit decline. A significant finding of this research is the successful substitution of logistic regression parameters with Shapley values to derive credit scores using the methodology outlined in [4], showcasing the practical applicability of Shapley values in credit scoring.

### Interpretable credit models–Home Credit data

Across the Home Credit data, Tables 16–26 illustrate the credit scores of the eleven predictor variables. Notably, in all instances, the five models consistently agree on which predictor variable bins fall below the neutral credit score, thus providing potential explanations for why customers might receive lower scores.

**Table 16:** Average–Approved annuity amount.

		Logistic Regression	XGBoost	Random Forest	LightGBM	CatBoost
**Predictor Variable**	**Bin**	**Credit Score**				
Average–Approved annuity amount	(-inf, 4160.83)	23.6388	104.1387	121.8600	107.3289	0.0000
	[4160.83, 8934.75)	91.5894	116.3997	121.8615	117.3830	26.1096
	[8934.75, inf)	162.1021	128.6020	121.8626	129.5077	142.7540
**Neutral Credit Score**		135.5539	123.9826	121.8622	125.0247	103.1746

**Table 17:** Maximum number of days (relative to the application date) on which a payment was made for previous instalments.

		Logistic Regression	XGBoost	Random Forest	LightGBM	CatBoost
**Predictor Variable**	**Bin**	**Credit Score**				
Maximum number of days (relative to the application date) on which a payment was made for previous instalments	[-19.50, inf)	121.8622	106.8595	121.8618	115.0803	114.3648
	(-inf, -19.50)	124.4529	125.0546	121.8622	128.6087	123.2026
**Neutral Credit Score**		124.0384	122.1440	121.8623	126.4446	121.7888

**Table 18:** Maximum–Approved credit amount.

		Logistic Regression	XGBoost	Random Forest	LightGBM	CatBoost
**Predictor Variable**	**Bin**	**Credit Score**				
Maximum–Approved credit amount	(-inf, 50954.04)	0.0000	0.0000	121.8580	100.4851	88.3454
	[50954.04, 898398.00)	123.4501	126.0522	121.8622	122.8748	123.8610
	[898398.00, inf)	138.4285	165.5761	121.8623	132.4269	142.7153
**Neutral Credit Score**		112.5787	117.2354	121.8618	121.5474	122.1043

**Table 19:** Average of external scores (1, 2 & 3).

		Logistic Regression	XGBoost	Random Forest	LightGBM	CatBoost
**Predictor Variable**	**Bin**	**Credit Score**				
Average of external scores (1, 2 & 3)	(-inf, 0.42)	121.8622	28.2460	83.5474	35.5794	0.0000
	[0.42, inf)	190.7930	175.5838	138.3170	180.3757	288.2603
**Neutral Credit Score**		177.4769	147.1210	127.7366	152.4039	232.5741

**Table 20:** Normalized score from external data source 3.

		Logistic Regression	XGBoost	Random Forest	LightGBM	CatBoost
**Predictor Variable**	**Bin**	**Credit Score**				
Normalized score from external data source 3	(-inf, 0.31)	121.8622	0.0000	92.8836	94.9131	0.0000
	[0.31, inf)	162.9762	215.6522	129.7101	132.4732	160.9843
**Neutral Credit Score**		156.9643	184.1183	124.3251	126.9810	137.4443

**Table 21:** Normalized score from external data source 2.

		Logistic Regression	XGBoost	Random Forest	LightGBM	CatBoost
**Predictor Variable**	**Bin**	**Credit Score**				
Normalized score from external data source 2	(-inf, 0.35)	121.8622	0.0000	46.7201	0.0000	0.0000
	[0.35, inf)	172.4943	355.6024	153.1185	339.8045	215.9150
**Neutral Credit Score**		161.6762	279.6242	130.3853	267.2016	169.7825

**Table 22:** Normalized score from external data source 1.

		Logistic Regression	XGBoost	Random Forest	LightGBM	CatBoost
**Predictor Variable**	**Bin**	**Credit Score**				
Normalized score from external data source 1	(-inf, 0.27)	121.8622	49.3349	121.8610	47.4972	117.9383
	[0.27, inf)	129.7068	216.1394	121.8623	197.6447	138.7986
**Neutral Credit Score**		129.1451	204.1939	121.8622	186.8921	137.3047

**Table 23:** Maximum—How many days before current application did client apply for credit bureau credit.

		Logistic Regression	XGBoost	Random Forest	LightGBM	CatBoost
**Predictor Variable**	**Bin**	**Credit Score**				
Maximum—How many days before current application did client apply for Credit Bureau credit	[-76.50, inf)	121.8622	80.3987	107.6043	87.7062	11.3244
	(-inf, -76.50)	125.7284	131.9663	123.8594	137.1557	136.2507
**Neutral Credit Score**		125.3388	126.7700	122.2214	132.1728	123.6623

**Table 24:** Average—Days past due of instalments.

		Logistic Regression	XGBoost	Random Forest	LightGBM	CatBoost
**Predictor Variable**	**Bin**	**Credit Score**				
Average—Days past due of Instalments	[0.08, inf)	121.8622	102.7606	115.5192	95.9194	92.8871
	(-inf, 0.08)	138.9704	252.5404	127.8514	238.8954	150.6132
**Neutral Credit Score**		130.6599	179.7826	121.8609	169.4427	122.5719

**Table 25:** How many days before the application the person started current employment.

		Logistic Regression	XGBoost	Random Forest	LightGBM	CatBoost
**Predictor Variable**	**Bin**	**Credit Score**				
How many days before the application the person started current employment	[6123.75, inf)	121.8622	87.3832	121.8599	87.3148	112.2891
	(-inf, 6123.75)	151.7564	168.3528	121.8692	157.0443	172.7415
**Neutral Credit Score**		130.4769	110.7163	121.8626	107.4089	129.7098

**Table 26:** Ratio of annuity amount / Credit amount.

		Logistic Regression	XGBoost	Random Forest	LightGBM	CatBoost
**Predictor Variable**	**Bin**	**Credit Score**				
Ratio of Annuity amount / Credit Amount	[0.05, inf)	121.8622	65.6892	109.3365	71.7789	108.1582
	(-inf, 0.05)	141.7548	160.1158	145.3041	177.0741	143.2001
**Neutral Credit Score**		129.9096	103.8887	123.8869	114.3752	122.3341

The consistent agreement across all models regarding which predictor variable input values fall below or above the neutral credit score demonstrates the robustness of our approach and reinforces the potential of Shapley values as a viable alternative to logistic regression parameters for deriving interpretable credit scores, as demonstrated in the Taiwan dataset. This finding further supports the applicability of the methodology outlined in [4] for a broader range of credit scoring models.

## Conclusion and future work

As noted in the literature, the limited transparency of advanced machine learning models has been a barrier to their widespread adoption in credit scoring due to regulatory requirements [14, 71]. However, our findings demonstrate that transparency need not be a barrier, as credit scores derived from Shapley values align closely with those derived from logistic regression models.

Our research establishes that Shapley values can effectively identify reasons for unfavourable credit reports, aligning with industry practices and providing a valuable tool for interpreting complex machine learning models. Furthermore, our research confirms previous findings [5, 70] that tree-based models like XGBoost and random forest outperform logistic regression in terms of accuracy, solidifying their efficacy in credit scoring.

Building upon these findings, future research should focus on the practical implementation of the proposed interpretability methods within real-world credit scoring scenarios. Additionally, investigating the potential of these methods to enhance the interpretability of other ensemble models in various applications would be a valuable avenue for further exploration.
