# Correction: A novel framework for enhancing transparency in credit scoring: Leveraging Shapley values for interpretable credit scorecards

**DOI:** 10.1371/journal.pone.0329901

**Published:** 2025-08-05

**Authors:** Rivalani Hlongwane, Kutlwano Ramaboa, Wilson Mongwe

The second author’s name is spelled incorrectly. The correct name is: Kutlwano Ramaboa. The correct citation is: Hlongwane R, Ramaboa K, Mongwe W (2024) A novel framework for enhancing transparency in credit scoring: Leveraging Shapley values for interpretable credit scorecards. PLoS ONE 19(8): e0308718. https://doi.org/10.1371/journal.pone.0308718

The name Kutlwano Ramabao appears incorrectly throughout the article. The correct name is Kutlwano Ramaboa.

## References

[1] Hlongwane R, Ramabao K, Mongwe W. A novel framework for enhancing transparency in credit scoring: Leveraging Shapley values for interpretable credit scorecards. PLoS One. 2024;19(8):e0308718. doi: 10.1371/journal.pone.0308718 39133710 PMC11318906

